# Early Abusive Relationships–Influence of Different Maltreatment Types on Postpartum Psychopathology and Mother-Infant Bonding in a Clinical Sample

**DOI:** 10.3389/fpsyt.2022.836368

**Published:** 2022-05-30

**Authors:** Julia Frohberg, Antje Bittner, Susann Steudte-Schmiedgen, Juliane Junge-Hoffmeister, Susan Garthus-Niegel, Kerstin Weidner

**Affiliations:** ^1^Department of Psychotherapy and Psychosomatic Medicine, Faculty of Medicine, Technische Universität Dresden, Dresden, Germany; ^2^Institute for Systems Medicine, Faculty of Human Medicine, Medical School Hamburg, Hamburg, Germany; ^3^Institute and Policlinic of Occupational and Social Medicine, Faculty of Medicine, Technische Universität Dresden, Dresden, Germany; ^4^Department of Child Health and Development, Norwegian Institute of Public Health, Oslo, Norway

**Keywords:** child maltreatment, child abuse, mother-infant bonding, postpartum, clinical sample, mother-baby unit

## Abstract

Postpartum psychopathology is a well-documented risk factor for impaired mother-infant bonding and thus child development. Increasingly, the focus of research in this area lies on maternal adverse childhood experiences that mothers bring into the relationship with their own baby, especially regarding the possible intergenerational transmission of traumatic experiences. Several studies showed that there is no direct link between child maltreatment and mother-infant bonding as one part of mother-child relationship, but that this link is mediated by postpartum psychopathology. To date, few studies examined differential effects between sexual, physical, and emotional abuse, and physical and emotional neglect, especially in a clinical sample. The aim of this study is to investigate whether the relationship between child maltreatment, psychopathology, and mother-infant bonding can be found for different forms of child maltreatment in patients of a mother-baby unit. Our sample consisted of 330 mothers of a mother-baby-unit in a psychosomatic clinic, who filled out self-report measures at time of admission. Mothers reported on maternal child maltreatment history with the Childhood Trauma Questionnaire, on current psychopathology with the Brief Symptom Inventory, and on mother-infant bonding with the Postpartum Bonding Questionnaire. Mediation analyses were performed with psychopathology as mediator, child maltreatment history as independent, and mother-infant bonding as dependent variable. There was no total effect of child maltreatment on mother-infant bonding. However, there were significant indirect effects of child maltreatment in general (ab = 0.09) and of the various forms of child maltreatment on mother-infant bonding via psychopathology (0.16 ≤ ab ≤ 0.34). The strongest effect was found for emotional abuse. After controlling for psychopathology, the direct effect of physical abuse on mother-infant bonding presented as a negative significant effect. This indicates that the more severe the physical abuse experienced, the better the self-reported bonding. A similar, but non-significant trend was found for sexual abuse. Our findings highlight the importance of assessing neglect forms of child maltreatment as well as abuse in women during the perinatal period. It further supports initial findings that different forms of child maltreatment can have differential effects on mother-infant bonding as one aspect of the mother-child relationship. Further research should include observational data to compare with self-report measures.

## Introduction

Forms of abuse and neglect endured as a child can have tremendous effects not only on developmental processes during childhood but also later in life (1). According to the World Health Organization (WHO), child maltreatment (CM) “includes all types of physical and/or emotional ill-treatment, sexual abuse, neglect, negligence”^1^. In general, five types of CM can be distinguished: sexual, physical, and emotional abuse, and physical and emotional neglect.

We can assume that approximately one out of three adults experienced some form of maltreatment in childhood (2, 3). In a large British longitudinal birth cohort study, 27% reported to have experienced any kind of maltreatment as a child (4). In representative samples of the German population, the following prevalence rates were found for at least low to moderate exposure to maltreatment: 12.5–13.9% for sexual abuse, 12.0–12.4% for physical abuse, 14.9–18.5% for emotional abuse, 41.6–48.4% for physical neglect, and 40.3–49.3% for emotional neglect (3, 5).

CM has been linked to negative outcomes on physical (e.g., neurological, gastrointestinal, cardiovascular, gynecological symptoms, and psychosomatic and pain syndromes) as well as mental health (e.g., depression, posttraumatic stress disorder, anxiety disorders, eating disorders, personality disorders, substance abuse, self-harming behavior, and suicidality) in later life (6). Exposure to CM in general has been associated with earlier age of onset in mental disorders, more severe symptoms and course, higher risk of suicide and comorbid mental disorders, and decreased quality of life (7). Differential effects of different forms of CM have been described in a recent review with studies focusing on physical and sexual abuse (8). An independently increased risk for anxiety disorders, depression and suicide attempts in patients with a history of sexual abuse was reported, as well as an independent association of reported physical abuse and illness severity and course in bipolar disorders (8). Emotional abuse and neglect seem to have strong association with characteristics of depressive disorders, independent from other CM forms (8). In all of this, however, it is important to note that the individual forms of CM rarely occur in isolation. Research showed that the more CM forms a child has been exposed to, the higher the risk for depression in adulthood and the higher the number of mental disorders (9, 10).

In addition to the impact on the life of CM victims themselves, research has focused heavily on the impact of CM on the parent-/mother-child relationship and a possible transgenerational transmission of CM risk (11).

The term mother-child relationship includes different facets such as bonding, attachment, interaction/parenting behavior, parental sense of competence and self-efficacy. These are not always clearly distinguished in the literature. According to a concept analysis, mother-infant bonding (MIB) is defined as an “affective state of the mother; maternal feelings and emotions toward the infant” (12). According to this definition, MIB is always a subjective emotional state of the mother. It can be assessed, for example, with the Postpartum Bonding Questionnaire (PBQ) (13). Attachment on the other hand is often understood as the bond from infant to mother (12). Interaction refers to the behavior displayed between mother and child, which is often assessed by observational methods (14).

The literature describes consequences of CM on different aspects of the mother-child relationship. Systematic reviews showed an association between childhood sexual abuse and later parenting stress (15), as well as tentative links between childhood emotional abuse and neglect and later self-reported parenting stress, dysfunctional interactions, and lower acceptance and empathy toward the child (16). Systematic reviews and meta-analyses also indicated a direct or indirect effect of childhood abuse on later observed caregiving behavior, e.g., maternal sensitivity, responsiveness, and intrusiveness (17, 18). Studies looking at the consequences for the children described that reduced maternal sensitivity and empathy are associated with a higher risk for mental disorders and reduced inhibition control in school-aged children (19–21).

Overall, these findings indicate a link between maltreatment experienced as a child and the later quality of the mother-child relationship. However, the picture of transgenerational transmission of CM risk is not clear (22). Several studies have looked at possible mediating factors like parenting young, mental health status, and maternal emotional dysregulation, thus questioning whether CM in childhood directly leads to problems in the mother-child relationship (23–25).

To sum up, there is some literature reporting negative, partly mediated effects of CM on various measures of the mother-infant relationship. Few studies have reported on MIB. It is well established that the formation of a healthy bond between mother and infant is an important developmental task in the first year postpartum (12). Brockington (26) highlights the effects of an impaired MIB, which can lead to rejection or anger toward the infant, increasing the risk of CM for the infant with long-term consequences for the mother-child relationship and child development. The overall literature shows a link between postpartum mental disorders and MIB disorders (27–29).

Some studies investigated the extent to which CM plays into this link. For example, Muzik et al. (30) showed that a history of maternal abuse and neglect was associated with greater MIB impairment (assessed with the PBQ) over the first 6 months postpartum. However, when modeled with current maternal psychopathology, this association was no longer significant. The authors conclude that it is not CM *per se*, but rather the resulting psychological symptoms that can have an impact on MIB. In another study, Muzik et al. (31) showed that depressed mothers reported the most severe bonding impairment compared to healthy or resilient mothers, regardless of comorbid PTSD symptoms following child abuse history.

The described studies used broad measures of CM, usually asking for any type of CM with the Childhood Trauma Questionnaire (CTQ) (32). Until now, only few studies examined differential effects of different CM types. Lang et al. (33) evaluated the impact of a history of sexual, physical, and emotional abuse (assessed with the abuse scales of the CTQ) on infant temperament and mother-child interaction, all self-reported, in a relatively healthy sample of women. However, MIB was not assessed. They found different response patterns for emotional and physical abuse, thus highlighting the importance of a closer look on maternal maltreatment history. Former emotional abuse in mothers led to more perceived interactional difficulties, but also lower levels of frustration perceived in infant temperament. Former physical abuse on the other hand was associated with an infant temperament perceived as more challenging, while reporting less interactional difficulties. Thus, in this study, physical abuse tended to have a counterintuitive positive effect on perceived mother-child interaction. Lehnig et al. (34) looked for specific effects of different CM types (also with the CTQ) on mother infant bonding, in a large sample and recruited from the general population. Physical and emotional neglect proved to be significant predictors for MIB, even after adding postpartum psychopathology as a predictor. While emotional neglect was shown to be associated with more MIB impairment, physical neglect was associated with less. Further, in a prospective longitudinal study examining a clinical sample of pregnant women with self-reported psychological symptoms (*n* = 251) it was shown that emotional abuse in childhood, among other factors, was a predictor for MIB impairment 6–7 weeks postpartum. However, physical and sexual abuse did not prove to be significant predictors (35). In this study, MIB was assessed with the PBQ and CM was assessed with the Early Trauma Inventory-Self Report (ETI-SR), which does not include forms of neglect.

In summary there are only few studies with partly surprising results investigating the differential implications of different types of CM on various aspects of the mother-child relationship. To our knowledge, no study has yet investigated the effect on bonding in a clinical sample of mothers with diagnosed postpartum mental disorders. The aim of this study is therefore to investigate whether the described relationship between CM, psychopathology, and MIB can be found for CM in general and different forms of CM in mothers treated in a day-care mother-baby-unit.

Based on the reviewed literature, we hypothesize, first, that the association between CM history in general and MIB is mediated in part by maternal psychopathology. Second, looking at the different forms of CM, we would expect there to be stronger effects for emotional abuse and emotional neglect, as emotional maltreatment has previously been shown to have an impact on MIB (34, 35) and our dependent variable is an emotional concept.

## Materials and Methods

### Participants and Procedure

We examined patients of a mother-baby unit at the Department of Psychotherapy and Psychosomatic Medicine of the University Hospital Dresden (Germany). Mothers are referred to the clinic mainly by outpatient clinicians (e.g., midwifes, psychotherapists, psychiatrists, obstetricians) or self-referred, usually as first treatment. Treatment is voluntary and is provided for mothers with postpartum psychiatric diagnoses and/or impaired mother-child bonding for whom outpatient treatment is not sufficient. Exclusion criteria for the treatment are acute suicidality or psychotic symptoms. Patients have to be at least 18 years old, treatment can usually be offered up to the first birthday of the child. The mother-baby unit serves urban and rural population.

The mothers in our sample were admitted from May 2010 to August 2021. All mothers were treated with their infants in a multimodal day-care setting including family therapy, video intervention therapy, group psychotherapy, cognitive behavioral therapy, medical treatment, and counseling on childcare (36). Data of 379 women were available for the indicated period. We only included patients in our study who were admitted to the mother-baby-unit for the first time and filled out the questionnaire at admission. Following these criteria, we excluded 20 mothers. Also, data for our analyzed variables was missing from 29 mothers, so in our final study sample we included *n* = 330 mothers. All included mothers gave written informed consent. Approval by the ethics committee of the Technische Universitaet Dresden was given for the study conduct (EK45022013).

Table 1 shows the sociodemographic characteristics of the final sample. On average, women were 30 years (SD = 5.9) old. Their infants were 23 weeks (SD = 12.6) old at time of admission, ranging from 2 to 76 weeks. About two thirds of the women (68.1%) were first-time mothers. Regarding education, 17.0% had graduated after 9 years of school, 35.5% after 10 years, and 40.9% had obtained their A-levels. Most of the mothers (about 80%) indicated that they were on maternity or parental leave. The majority lived in a partnership (70.3%), but was not married (62.4%). According to demographic statistics, this roughly corresponds to the general population in Eastern Germany (Saxony): about 40% of women aged 20–40 are married^2^. In about 60% of the children born, the parents are not married^3^. When asked about household income, about 20% reported a household income of less than 1,000 euros, about 30% reported a household income between 1,000 and 2,000 euros, and about 38% reported a household income of more than 2,000 euros.

**Table 1:** Sociodemographic characteristics of the sample (*n* = 330).

Age; M (SD)	29.7 (5.9)
Current life situation	
With partner; *n* (%)	232 (70.3%)
With partner, but without joint household; *n* (%)	22 (6.7%)
Single; *n* (%)	43 (13.0%)
Other; *n* (%)	23 (7.0%)
Missing values; *n* (%)	10 (3.0%)
Marital status	
Unmarried; *n* (%)	206 (62.4%)
Married; *n* (%)	96 (29.1%)
Divorced; *n* (%)	16 (4.8%)
Widowed; *n* (%)	1 (0.3%)
Missing values; *n* (%)	11 (3.4%)
Education	
Graduation after 9 years of schooling; *n* (%)	56 (17.0%)
Graduation after 10 years of schooling; *n* (%)	117 (35.5%)
A-Level; *n* (%)	135 (40.9%)
No graduation; *n* (%)	12 (3.6%)
Missing values; *n* (%)	10 (3.0%)
Parity	
Primiparous; *n* (%)	194 (68.1%)^a^
Age of childre*n* (in weeks) at admission; M (SD)	23.3 (12.6)
Household income	
<500 Euro; *n* (%)	21 (6.4%)
500–999 Euro; *n* (%)	45 (13.6%)
1,000–1,499 Euro; *n* (%)	42 (12.7%)
1,500–1,999 Euro; *n* (%)	58 (17.6%)
2,000–2,500 Euro; *n* (%)	52 (15.8%)
>2,500 Euro; *n* (%)	75 (22.7%)
Missing values; *n* (%)	37 (11.2%)

*M, mean; SD, standard deviation*.

a *Percentage calculated from valid cases*.

**Figure 1:**
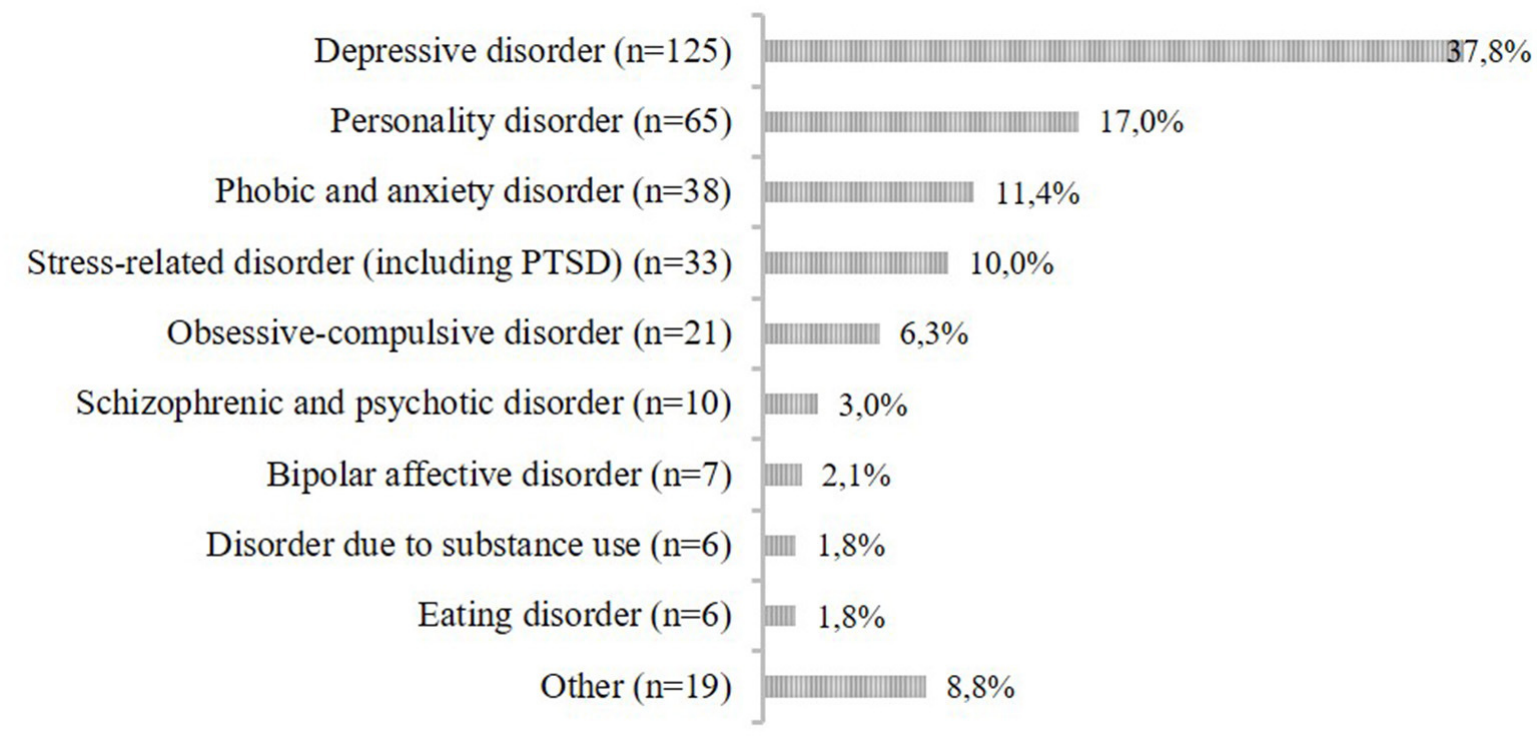
Primary diagnoses of the sample (*n* = 330).

Diagnoses were made on the basis of a comprehensive clinical assessment including a structured clinical interview for DSM-IV (SCID) (37). Figure 1 shows the primary diagnoses of our sample. Most common were depressive disorders (37.8%), personality disorders (17.0%; dissocial, emotionally unstable, histrionic, anankastic, anxious, and dependent personality disorders) and anxiety disorders (11.4%; panic disorder, phobic anxiety disorders, other anxiety disorders, obsessive-compulsive disorders, reaction to severe stress, dissociative disorders, and somatoform disorders). The majority of our sample had comorbid mental disorders (71.5%). When looking at neurotic and stress-related disorders, 24 women (7.3%) had posttraumatic stress disorder (PTSD) as a primary diagnosis, 33 additional women had it as a secondary diagnosis, with various underlying traumas.

### Measures

Self-report measures were completed within the first 2 weeks after admission to the mother-baby-unit.

Maternal CM was assessed retrospectively with the Childhood Trauma Questionnaire (CTQ) (32). It consists of 28 items, rated on a 5-point Likert scale (*never true* to *very often true*) and differentiates between five types of maltreatment: sexual abuse, physical abuse, emotional abuse, physical neglect, and emotional neglect. In addition, there are three items that indicate minimizing tendencies. Scores for each scale can be categorized by severity: none to low trauma exposure, low to moderate trauma exposure, moderate to severe trauma exposure, and severe to extreme trauma exposure. Examination of the psychometric properties of the German version showed high internal consistency and good validity, similar to the English original (38). The CTQ has been used before in clinical samples of mothers with perinatal psychological problems [e.g., (31)]. For the analyses, we used the total score, ranging from 0 to 125, as well as a scale score for each type of maltreatment, ranging from 0 to 25. Higher scores indicate more severe maltreatment exposure. The Cronbach alpha for the total score in this clinical sample was 0.93, the Cronbach alpha for the CTQ subscales ranged from 0.67 (physical neglect) to 0.95 (sexual abuse).

Mothers provided information on current psychopathology with the Brief Symptom Inventory (BSI), a short form of the SCL-90 (39). It consists of 53 items, which are answered on a 5-point Likert scale (*not at all* to *very much*). For the analyses, we used the Global severity index (BSI-GSI), a sum score reflecting overall psychological distress. The cut-off for significant psychological distress is 0.62 for women (40). The German version has good psychometric properties, with excellent reliability for the BSI-GSI (Cronbach's alpha = 0.96) (41). The Cronbach alpha was 0.96 for the current sample.

Mothers reported on MIB with the Postpartum Bonding Questionnaire (PBQ). The PBQ was developed by Ian Brockington to provide an easy-to-use screening questionnaire to identify disorders in MIB (13, 42) and is well established in research and practice. It consists of 25 items, answered on a six-point Likert scale (*always* to *never*). The original version suggested a four factor solution (general factor for impaired bonding; rejection and pathological anger; infant-focused anxiety; incipient abuse), which could not be confirmed for the German version (43, 44). Reck et al. (43) found only low internal consistency for the scales infant-focused anxiety and incipient abuse. Therefore, for this study we calculated a sum score for the 25 items, with scores above 25 indicating the presence of a bonding disorder and scores above 39 indicating severe bonding disorders (42). A similar approach is described by Bittner et al. (45). The Cronbach alpha for this sum score was 0.95 in our sample.

### Statistical Analyses

**Figure 2:**
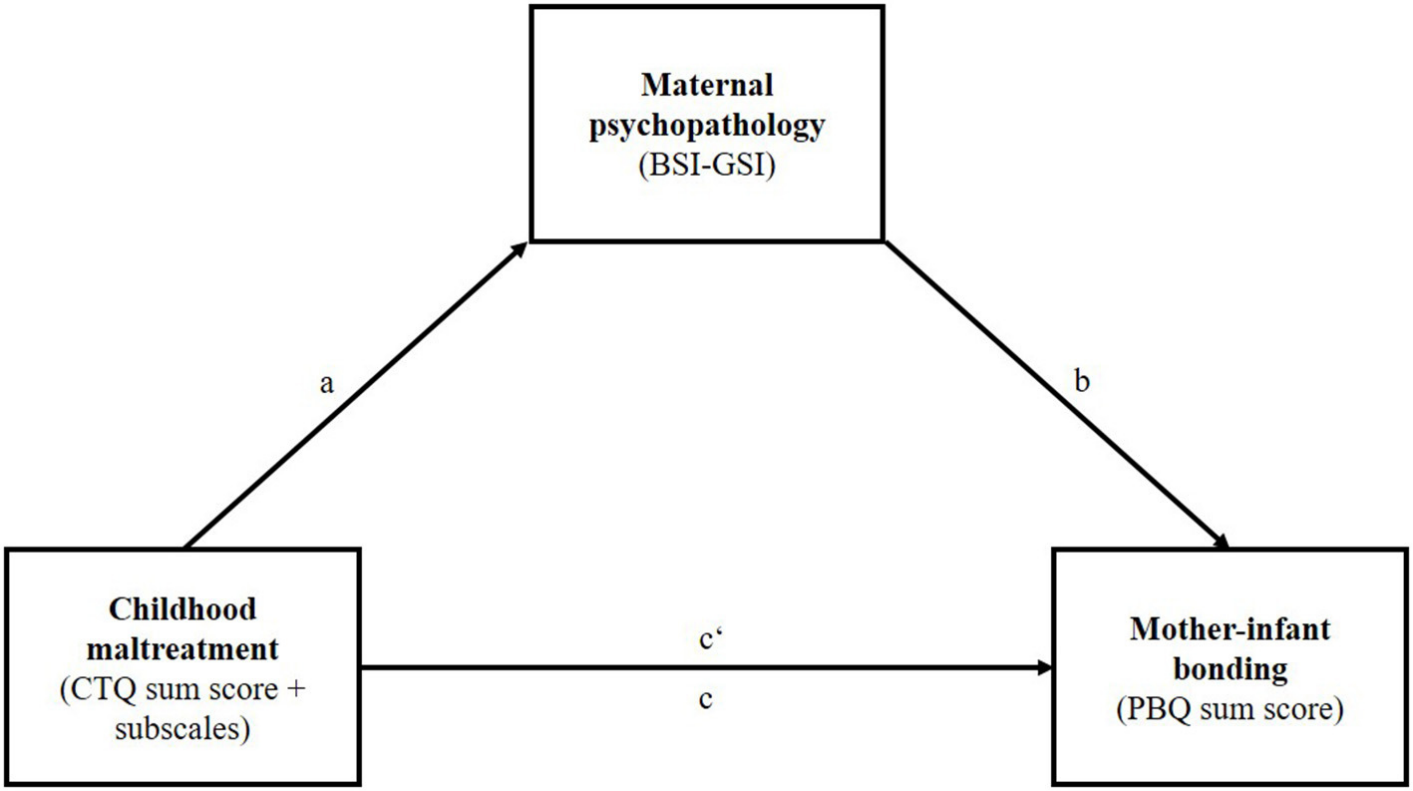
Schematic depiction of the analyzed mediation model.

We used SPSS for Windows, version 27 (IBM, Chicago, Illinois), for descriptive analyses, to calculate means, standard deviations, frequencies, Pearson correlation coefficients, and *t*-tests. For unequal variances, the Welch test was used. We used Hayes' PROCESS macro on SPSS (46), to perform separate mediation analyses, with the CTQ total score and each of the CTQ scales as independent variable, PBQ sum score as dependent variable, and BSI-GSI as mediator variable. Figure 2 shows our mediation model. For the mediation analyses 5,000 bootstrapping samples and 95% confidence intervals were used. Confidence intervals not including 0 suggest a significant indirect effect. A complementary adjustment for multiple testing was performed using the Holm-Bonferroni correction (47). For supplementary analyses we included covariates in the mediation analyses.

## Results

### Descriptive Analyses

In our sample, 83.3% reported at least low to moderate trauma exposure on at least one of the CTQ scales, with 12.7% of our participants reporting one type of CM, 21.5% reporting two types, 18.2% reporting three, 18.8% reporting four types, and 12.1% reporting exposure to all five types of CM assessed by the CTQ. Exposure to emotional neglect (71.2%) end emotional abuse (63.9%) were reported most frequently by all mothers, followed by exposure to physical neglect (49.4%), sexual abuse (33.6%), and physical abuse (27.9%).

Table 2 shows the descriptive statistics and intercorrelations of the variables used for analysis. The mean sum score for the Global Severity Index of the BSI was 1.25 (SD = 0.67) indicating high psychological distress. The mean PBQ sum score (*M* = 31.08; SD = 21.64) was also high, indicating the presence of bonding disorders in our sample. However, standard deviation was high too, indicating a great variance in relation to MIB disorders. The mean sum scores for the CTQ subscales indicated low to moderate severity of CM. It should be noted that here standard deviation was high as well, it tended to encompass one to two severity categories.

**Table 2:** Descriptive statistics and intercorrelations for all variables under study (*n* = 330).

		**Intercorrelations**						
**Measures**	**Mean** **±SD**	**(1)**	**(2)**	**(3)**	**(4)**	**(5)**	**(6)**	**(7)**
(1) BSI: Global Severity Index	1.25 ± 0.67							
(2) PBQ–Sum score	31.08 ± 21.64	0.23^***^						
(3) CTQ–Total score	48.19 ± 18.40	0.29^***^	−0.00					
(4) CTQ–Sexual abuse	7.11 ± 4.54	0.14^*^	−0.07	0.64^***^				
(5) CTQ–Physical abuse	7.28 ± 3.93	0.21^***^	−0.07	0.76^***^	0.45^***^			
(6) CTQ–Emotional abuse	11.86 ± 5.63	0.37^***^	0.06	0.87^***^	0.40^***^	0.62^***^		
(7) CTQ–Physical neglect	8.58 ± 3.65	0.17^**^	−0.04	0.81^***^	0.37^***^	0.55^***^	0.61^***^	
(8) CTQ–Emotional neglect	13.36 ± 5.51	0.24^***^	0.06	0.85^***^	0.34^***^	0.47^***^	0.72^***^	0.71^***^

*SD, standard deviation*.

* *p <0.05*;

** *p <0.01*;

*** *p <0.001*.

**Figure 3:**
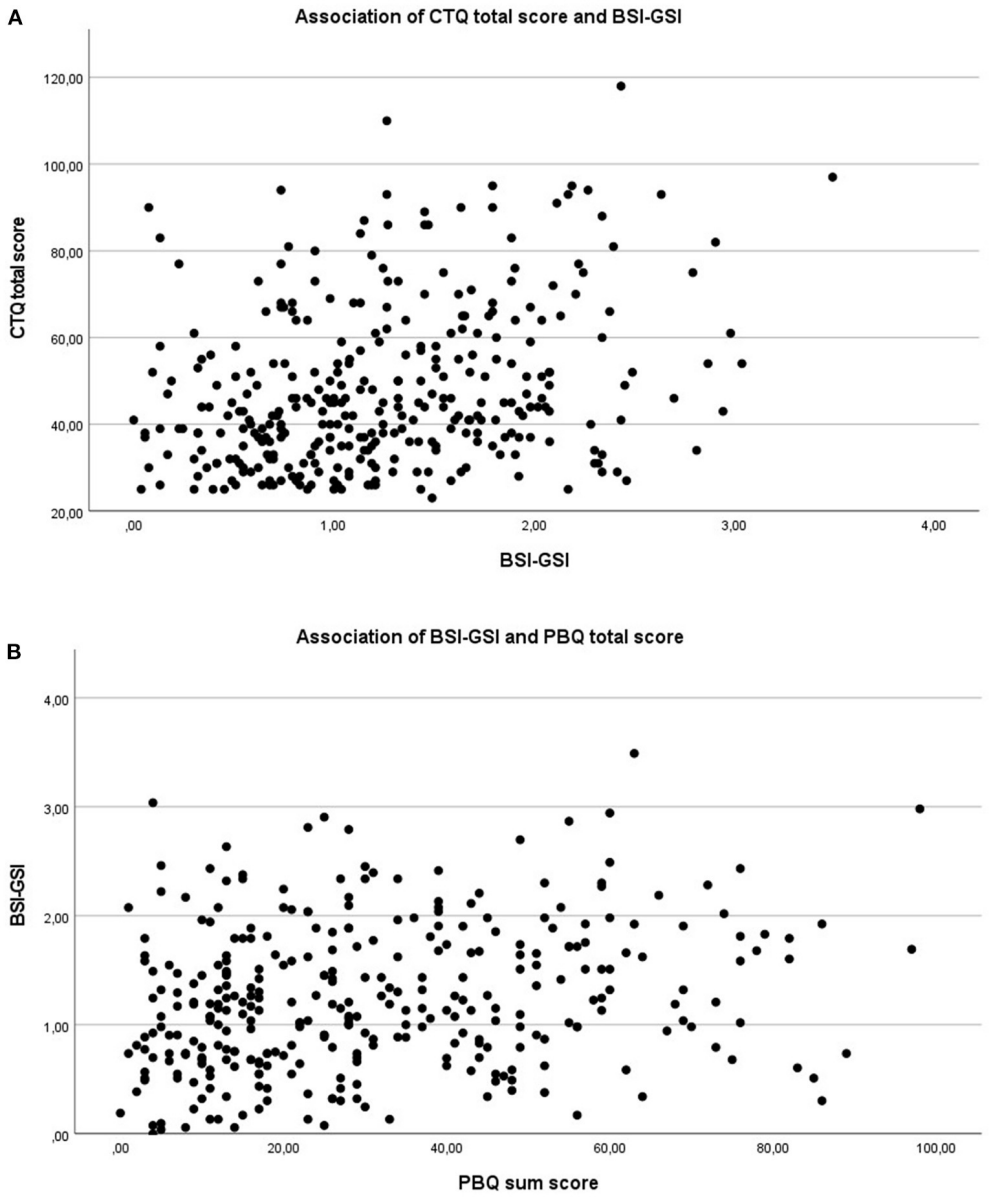
Scatterplots for the association between CM/psychopathology and psychopathology/MIB. **(A)** Association of CTQ total score and BSI-GSI; *r* = 0.29, *p* < 0.001. **(B)** Association of BSI-GSI and PBQ sum score; *r* = 0.23, *p* < 0.001.

We found weak to medium significant correlations between the CTQ variables and the BSI-GSI (0.14 ≤ r ≤ 0.37; *p*' <0.014) as well as the BSI-GSI and PBQ sum score (*r* = 0.23; *p* < 0.001), depicted in Figure 3. Looking at the CTQ, we found strong correlations between the total score and the scales (0.64 ≤ r ≤ 0.87; *p*' <0.001) and medium to strong correlations in-between the five scales of the CTQ (0.34 ≤ r ≤ 0.72; *p*' <0.001). There was no significant correlation between the PBQ sum score and the scales or total score of the CTQ (*p*' > 0.226).

When looking at mean differences, we found that for the BSI-GSI, participants who reported at least low to moderate maltreatment exposure scored significantly higher than participants who reported none to low maltreatment exposure. This significant result was found for all subscales (*p*' <0.003), except for physical neglect (*p* = 0.066). There was also a significant difference in participants who experienced at least low to moderate maltreatment exposure on at least one subscale of the CTQ and parcipitants, who reported no exposure to any maltreatment (*p* < 0.001). Thus, the patients with childhood maltreatment experienced significantly higher psychological distress. When looking at the PBQ sum score, we found that participants who reported at least low to moderate exposure to emotional neglect scored significantly higher than participants who reported none to low exposure to emotional neglect (*p* = 0.002), indicating. There was no significant mean difference in PBQ sum score for the other subscales of the CTQ (*p*' > 0.079). Thus, there were no differences in reported MIB between patients who experienced sexual abuse, physical abuse, emotional abuse, and physical neglect, and patients who did not. Also, participants who experienced at least low to moderate maltreatment exposure on at least one subscale of the CTQ reported significant more impairment in MIB than participants who reported no exposure to any maltreatment (*p* = 0.028).

### Mediation Analyses

Table 3 shows the results of the mediation analyses for the total score of the CTQ as well as the five different types of CM assessed with the CTQ.

**Table 3:** Results of the mediation analyses for CM on MIB with psychopathology as mediator (*n* = 330).

**CTQ**	**Total effect of X on Y (c)**	**Effect of X on M (a)**	**Effect of M on Y (b)**	**Direct effect (c')**	**Indirect effect (ab)^a^**
Total score	−0.00	**0.01** ^***^	**8.29** ^***^	**–**0.09	0.09 95%-CI [0.04, 0.14]
Sexual abuse	−0.32	0.02*	**8.00** ^***^	−0.48	0.16 95%-CI [0.01, 0.35]
Physical abuse	−0.36	**0.04** ^**^	**8.38** ^***^	−0.66^*^	0.29 95%-CI [0.09, 0.57]
Emotional abuse	0.22	**0.04** ^***^	**7.93** ^***^	−0.12	0.34 95%-CI [0.16, 0.54]
Physical neglect	−0.21	**0.03** ^**^	**7.96** ^***^	−0.45	0.24 95%-CI [0.07, 0.45]
Emotional neglect	0.24	**0.03** ^***^	**7.51** ^***^	0.03	0.22 95%-CI [0.09, 0.38]

* *p <0.05*;

** *p <0.01*;

*** *p <0.001*.

*Bolded effect sizes indicate a significant result after adjusting for multiple testing with Holm-Bonferroni correction*.

a *Indirect effect is significant when the 95% CI does not contain zero*.

When looking at the effects of CM history in general, we did not find a significant total effect (c) of the CTQ total score on MIB (*p* = 0.978). However, we found a significant effect of the CTQ total score on current psychopathology (path a) (*p* < 0.001), which in turn had a significant effect on MIB (path b) (*p* < 0.001). There was also evidence of a small significant indirect effect (ab = 0.09, 95%-CI [0.04, 0.14]). When controlling for current psychopathology, the direct effect (c) remained non-significant (*p* = 0.173).

In the next step, we looked at differential effects of different CM types. First, we did not find a significant total effect (c) of any CM type on MIB (*p*' > 0.230). Second, we found significant relations between CM history and current psychopathology (path a) (*p*' <0.014) for all CTQ scales. The largest effect was shown by emotional abuse on psychopathology (*B* = 0.04, SE = 0.006, *t* = 7.09, *p* < 0.001), the smallest effect was shown by sexual abuse (*B* = 0.02, SE = 0.008, *t* = 2.47, *p* = 0.014).

Third, we found significant relations between current psychopathology and MIB (path b) (*p*' <0.001) for all CTQ scales. The largest effect was shown when entering physical abuse as independent variable (*B* = 8.38, SE = 1.77, *t* = 4.73, *p* < 0.001), the smallest effect was shown when entering emotional neglect as independent variable (*B* = 7.51, SE = 1.80, *t* = 4.18, *p* < 0.001).

After adjusting *p*-values for multiple testing with the Holm-Bonferroni correction, relations between CM history and current psychopathology remained significant (adjusted *p*' <0.038), except for sexual abuse (adjusted *p* = 0.182). Relations between current psychopathology and MIB (path b) also remained highly significant (adjusted *p*' <0.001).

For all types of CM, there was evidence of significant indirect effects (ab), suggesting that the relationship between CM and MIB was mediated by maternal psychopathology. The largest effect here was found for emotional abuse (ab = 0.34, 95%-CI [0.16, 0.54]), the smallest effect for sexual abuse (ab = 0.16, 95%-CI [0.01, 0.35]).

After entering the mediator into the model, the direct effect (c') of emotional abuse, physical neglect, and emotional neglect on MIB was still not significant, regardless of psychopathology. However, for physical abuse (*B* = −0.66, SE = 0.30, *t* = −2.21, p=0.028), the direct effect became statistically significant and presented as a negative effect. Those results suggest that, when controlling for current psychopathology, the more severe the physical abuse experienced, the better the self-reported MIB. A similar, but only nearly significant pattern was found for sexual abuse (*B* = −0.48, SE = 0.25, *t* = −1.86, *p* = 0.065). This direct negative effect for physical abuse became non-significant after applying a Holm-Bonferroni correction (adjusted *p* = 0.335).

Supplementary analyzes with possible covariates showed that the mother's age and professional training had no significant effect on the mediation model. For household income and educational level of the mother, a significant relationship to MIB was found. Higher socioeconomic status was associated here with more self-reported bonding issues. However, this did not change the basic results of the mediation analysis. The indirect effects found were stable even when these covariates were included in the model.

## Discussion

The purpose of this study was to investigate the association between CM, maternal psychopathology, and MIB. The focus was on the effects of different forms of CM, as well as assessing MIB as a specific aspect of the mother-child relationship. This was examined in a clinical sample of mothers suffering from postpartum mental disorders receiving treatment at a day-care mother-baby unit.

To the best of our knowledge, there has only been one study using a similar approach in a general population sample (34) and one study in a clinical sample, that assessed only the abuse types of maltreatment (35).

Reported CM in our sample was high, as would be expected in a clinical context (48). About 80% reported exposure to at least one type of trauma or neglect, which is higher than in the general population (5). The most common types of CM in our sample were emotional abuse and emotional neglect. This is in contrast to representative German samples, where the most common types of CM were physical and emotional neglect (3, 5).

First, we did not find a total effect of any of the CM types or CM history in general on MIB. When entering the mediator psychopathology into the model, we found a significant pattern similar for all CM types: the more severe the reported maltreatment, the higher the psychological distress and the higher the psychological distress, the greater the perceived impairment in MIB. We also found significant indirect effects suggesting that the association between CM and MIB was partly mediated by maternal psychopathology. This is in line with findings that not the CM itself but the resulting psychopathology in postpartum mothers affects MIB (30, 31). Further research should clarify what type of psychopathology is involved in mediation. To date, studies seem to focus on depressive symptoms (23, 27, 31). Studies that also included anxiety sometimes showed an effect of anxiety on MIB (35, 49) and sometimes did not (34).

Second, when focusing on the differential effects of various CM forms, we found the strongest effect for emotional abuse. This is consistent with other findings highlighting the role of emotional maltreatment on MIB impairment (34, 35). In the literature the association between emotional maltreatment history and deficits in different aspects of mother-child relationship is explained by the forming of maladaptive relationship schemas in childhood, like seeing others as hurtful or neglectful (16, 50).

We found this mediation effect of psychopathology regardless of the type of maltreatment, suggesting that this association can also be also found for physical and emotional neglect. The effect of these forms on MIB has hardly been studied so far. This finding highlights the importance of assessing less obvious forms of maltreatment, such as neglect, when looking at trauma history in pregnant women.

Surprisingly, the direct effect of physical abuse on MIB presented as a negative significant effect, implying that the more severe the physical abuse experienced, the less impairment in MIB was reported, when controlling for maternal psychopathology. A similar pattern was found for sexual abuse, but the direct effect here was a non-significant trend. It should be noted that this effect did not remain significant when adjusting for multiple testing with the Holm-Bonferroni correction. As there are only few studies looking at differential effects of different CM types we still want to discuss this finding with some caution. Lehnig et al. (34) found a similar counterintuitive effect for physical neglect, showing that mothers who reported more severe physical neglect reported less impairment in MIB. However, this also should be interpreted with caution due to a small Cronbach's alpha of this subscale in their study. As a possible explanation, the authors name the desire of the women to compensate harmful relationships experienced as a child.

For emotional abuse, physical neglect, and emotional neglect, direct effects remained nonsignificant. One possible explanation for this could be that physical and sexual abuse are more “obvious,” “socially recognized” forms of CM and therefore more easily recognized by the victims themselves. This could be a prerequisite for wanting opposite experiences in the relationship with one's own child and deliberately developing positive attitudes toward parenting. This may be supported by the finding that in mothers with a history of CM the majority reported positive parenting-specific posttraumatic changes (51). Posttraumatic changes are defined as “alterations in views of the self, relationships, or world that result from efforts to make meaning of CM experiences” (51). It would also be interesting to consider the extent to which psychotherapy influences these posttraumatic changes by reflecting on biographical experiences and correcting dysfunctional relational experiences. Further research should again distinguish between different types when looking at these posttraumatic changes in parenting views.

Thus, the effect we found for physical abuse (and the trend for sexual abuse) may reflect more of a desire for a healed relationship in this context. Because we examined a self-rated concept, it could also be influenced by social desirability. These findings on MIB should therefore be distinguished from actual interaction behavior. For example, Noorlander et al. (52) showed that there were almost no significant correlations between self-reported MIB and observed interaction at the time of admission in a small sample of depressed mothers. However, there are also findings of significant associations between the two (14). Thus, to determine whether CM has an effect on mother-child interaction, we have to look at observational data. To date, there are mixed results regarding this and again, only few studies differentiate between different CM types. Tambelli et al. (53) found that mothers with a history of physical/sexual abuse as well as emotional abuse/neglect showed more maladaptive interactional behavior in an observed feeding situation than mothers with no history of CM. It was also shown that maternal CM leads to problems in mother-infant dyadic reciprocity mediated by lower maternal attention to infant faces (54), and that it leads to less sensitive behavior in mothers mediated by parenting stress (55). Another study found no connection between a history of CM and less positive or more negative parenting behavior (56).

## Strengths and Limitations

The strength of this study lies in its exploration of a broad spectrum of CM in a large clinical sample, therefore taking a closer look at possible differences in the effects of different maltreatment types and including neglect forms of CM as well as abuse. Based on a clinical care sample of a specialized treatment program for mothers with postpartum mental disorders, we also examined maternal psychopathology influencing MIB.

However, there are also several limitations with respect to our sample. Because this was a clinical care sample the study variables were not assessed at a standardized postpartum time frame, times of admission varied from 2 to 76 weeks postpartum. However, the majority of the sample was admitted in the first year postpartum (only six women were admitted between 53 and 76 weeks postpartum). Also, some of the participants underwent psychotherapeutic sessions at our perinatal outpatient department or were already in therapy prior to admission. Thus, it is possible that treatment effects on MIB were already in effect for part of our sample.

We used only self-report questionnaires, even if they were well established and widely used. However, responses could be influenced by social desirability or lack of parenting self-confidence (57). Almost half of our sample had a depression or anxiety disorders. Those emotional disorders are associated with negatively biased interpretation of ambiguous situations as well as increased memory of negative experiences (58). Thus, this could influence the reporting of experienced maltreatment in the CTQ. On the other hand studies showed that CM ratings remained stable even when self-reported symptoms decreased, indicating that CM ratings are not strongly influenced by current depressive symptoms (59, 60).

In our study, the CTQ scales were considered independently. Thus, possibly existing interaction effects between the scales were neglected, even though 70% of our sample reported exposure to more than one type of CM. It seems plausible that the different forms of CM often occur together (61). Intercorrelations of the CTQ scales in our study varied between medium to strong correlations and were similar to those of a German validation sample for the CTQ (38).

Finally, it should be noted that CM in our study was only retrospectively assessed as part of a cross-sectional study. This can lead to memory distortions (62). Bernstein et al. (32) showed good criterion validity in the development of the CTQ, and a more recent study found high convergent validity in a perinatal sample (63). Nevertheless, a review by Georgieva et al. (64) concluded that further research on criterion validity for the CTQ is needed. Longitudinal studies that collect data from childhood through own motherhood would be helpful here. However, conducting such studies is difficult for feasibility reasons.

In future research, we want to focus on the effect of CM on psychotherapeutic outcome in a day-care mother-baby-unit, also adding observational data on mother-child interaction to self-report-measures.

## Conclusion

Our findings indicate that the more severe the CM experienced the more severe the psychopathology, and the more severe the psychopathology the greater the perceived MIB impairment. We also found that the connection between CM and MIB is for a small part mediated over maternal psychopathology. This was found regardless of the type of maltreatment, showing that physical and emotional neglect have an effect on bonding as well as former child abuse. This underlines the importance of assessing a full trauma history for all perinatal women. Our findings are consistent with the literature that not the CM history itself, but the possible resulting postpartum psychopathology explains impaired MIB, showing that resilience plays an important role. Future research should review these findings on different forms of CM not only for self-report measures of bonding but also for observed interaction between mother and infant.

## Data Availability Statement

The dataset analyzed during the current study is not publicly available due to legal and ethical constraints, as the study's informed consent did not include public sharing of participant data. The dataset is available from the corresponding author on reasonable request. Requests to access the datasets should be directed to julia.frohberg@ukdd.de.

## Ethics Statement

The studies involving human participants were reviewed and approved by the Ethics Committee of the Technische Universitaet Dresden (EK45022013). The patients/participants provided their written informed consent to participate in this study.

## Author Contributions

JF, AB, and KW made substantial contributions to the conception and design of the present study. JJ-H was involved in study design and assessment of the clinical sample. JF analyzed the data and took the lead in writing the manuscript, while AB was supervising the project. AB, SS-S, SG-N, JJ-H, and KW revised the manuscript critically for important intellectual content. All authors contributed to the article and approved the submitted version.

## Funding

We acknowledge support by the open access publication funds of the SLUB/Technische Universität Dresden.

## Conflict of Interest

The authors declare that the research was conducted in the absence of any commercial or financial relationships that could be construed as a potential conflict of interest.

## Publisher's Note

All claims expressed in this article are solely those of the authors and do not necessarily represent those of their affiliated organizations, or those of the publisher, the editors and the reviewers. Any product that may be evaluated in this article, or claim that may be made by its manufacturer, is not guaranteed or endorsed by the publisher.

## Footnotes

^1^ www.who.int/health-topics/violence-against-children#tab=tab_2

^2^ www.statistik.sachsen.de/download/bevoelkerung/statistik-sachsen_aI_zr_ bevoelkerung-familienstand.xlsx

^3^ www.statistik.sachsen.de/download/statistische-berichte/bericht_statistik- sachsen_a-II-3_eheschliessungen-geborene-gestorbene.xlsx
